# Dose-dependent effects of GAT107, a novel allosteric agonist-positive allosteric modulator (ago-PAM) for the α7 nicotinic cholinergic receptor: a BOLD phMRI and connectivity study on awake rats

**DOI:** 10.3389/fnins.2023.1196786

**Published:** 2023-06-23

**Authors:** Brittany M. Brems, Erin E. Sullivan, Jenna G. Connolly, Jingchun Zhang, Arnold Chang, Richard Ortiz, Lucas Cantwell, Praveen Kulkarni, Ganesh A. Thakur, Craig F. Ferris

**Affiliations:** ^1^Department of Pharmaceutical Sciences, Northeastern University, Boston, MA, United States; ^2^Center for Translational Neuroimaging, Northeastern University, Boston, MA, United States; ^3^Department of Chemistry and Biochemistry, New Mexico State University, Las Cruces, NM, United States; ^4^Department of Psychology, Northeastern University, Boston, MA, United States

**Keywords:** somatosensory cortex, schizophrenia, resting state functional connectivity, basal ganglia, pain, corticothalamic neural circuitry

## Abstract

**Background:**

Alpha 7 nicotinic acetylcholine receptor (α7nAChR) agonists have been developed to treat schizophrenia but failed in clinical trials due to rapid desensitization. GAT107, a type 2 allosteric agonist-positive allosteric modulator (ago-PAM) to the α7 nAChR was designed to activate the α7 nAChR while reducing desensitization. We hypothesized GAT107 would alter the activity of thalamocortical neural circuitry associated with cognition, emotion, and sensory perception.

**Methods:**

The present study used pharmacological magnetic resonance imaging (phMRI) to evaluate the dose-dependent effect of GAT107 on brain activity in awake male rats. Rats were given a vehicle or one of three different doses of GAT107 (1, 3, and 10 mg/kg) during a 35 min scanning session. Changes in BOLD signal and resting state functional connectivity were evaluated and analyzed using a rat 3D MRI atlas with 173 brain areas.

**Results:**

GAT107 presented with an inverted-U dose response curve with the 3 mg/kg dose having the greatest effect on the positive BOLD volume of activation. The primary somatosensory cortex, prefrontal cortex, thalamus, and basal ganglia, particularly areas with efferent connections from the midbrain dopaminergic system were activated as compared to vehicle. The hippocampus, hypothalamus, amygdala, brainstem, and cerebellum showed little activation. Forty-five min post treatment with GAT107, data for resting state functional connectivity were acquired and showed a global decrease in connectivity as compared to vehicle.

**Discussion:**

GAT107 activated specific brain regions involved in cognitive control, motivation, and sensory perception using a BOLD provocation imaging protocol. However, when analyzed for resting state functional connectivity there was an inexplicable, general decrease in connectivity across all brain areas.

## 1. Introduction

The nicotinic acetylcholine receptor (nAChR) is an ion channel prevalent throughout the peripheral and central nervous systems. It exists as a pentamer containing any variety of alpha (α), beta (β), gamma (γ), or delta (δ) subunits (Albuquerque et al., 2009; Millar and Gotti, 2009) to form a central pore for the conductance of Na^+^, K^+^, or Ca^2+^ ions upon activation (Wong et al., 2014). There are 8 α-, 3 β-, 1 γ-, and 1 ς- subunit subtypes (Wooltorton et al., 2003; Dani, 2015). Binding of the endogenous ligand, acetylcholine (ACh), at the interface of two alpha subunits or between an α and β subunit results in activation and opening of the pore (Dani, 2015; Gulsevin et al., 2019). Once opened, Na^+^ and Ca^2+^ move into the cell while K^+^ moves out, resulting in the opening of voltage-gated ion channels to initiate an action potential and inducing activation of various Ca^2+^-dependent second-messenger cascades. The central nervous system nAChRs only consist of α and β subunits, with the two most prevalent subtypes being α7 and α4β2 (Dani, 2015).

α7 ligands have been shown to affect neuropathic pain, inflammation, and cognition (Wong et al., 2014) with high concentrations found in the hippocampus, frontal cortex, hypothalamus, and midbrain (Skoubis et al., 2006; Maier et al., 2011). Previously, α7 nAChR agonists have been tested for the treatment of schizophrenia, but failed in phase 3 clinical trials because of unwanted side effects (Tregellas et al., 2011; Tregellas and Wylie, 2019), and rapid receptor desensitization (Gulsevin et al., 2019). However, the positive allosteric modulators (PAMs) are promising therapeutics because they modulate the receptor function by binding to a site topographically distinct from the orthosteric site which can reduce the rapid desensitization. As a result, these are becoming the norm for creating drugs for this receptor. PAMs exist as either type 1 or type 2. Type 1 PAMs increase the peak current, or amount of cation influx, but do not alter the rate of desensitization, while type 2 PAMs increase the peak current and slow down desensitization (Bagdas et al., 2016).

GAT107 is an allosteric agonist-positive allosteric modulator (ago-PAM) for the α7 nAChR, meaning it can activate the receptor with or without a bound orthosteric ligand (Thakur et al., 2013; Bagdas et al., 2016; Horenstein et al., 2016) and also act as a PAM of orthosteric agonist. It is classified as a Type 2 PAM because it slows desensitization, as evident in electrophysiology studies (Horenstein et al., 2016; Miller et al., 2020). While GAT107 has not been tested in preclinical models of schizophrenia, is has been shown to be antinociceptive in the complete Freund’s adjuvant model of peripheral pain and able to block conditioned place aversion following an intrathecal acetic acid injection, suggesting it could be used to treat neuropathic pain (Bagdas et al., 2016). This analgesic effect may be partly due to GAT107’s role in the regulation of neuroinflammation by the downregulation of synthesis and release of inflammatory cytokines and attenuation of oxidative stress along with increasing the concentrations of the anti-inflammatory cytokine, IL-10 (Bagdas et al., 2016; Gauthier et al., 2021; Mizrachi et al., 2021). These different *in vivo* neurobiological effects may primarily be mediated by α7 nAChR activation by GAT107, or secondary activation of other signaling pathways.

The present study used pharmacological MRI (phMRI) to evaluate the dose-dependent effect of GAT107 on brain activity in awake rats. phMRI offers a method for screening GAT107 for its global activity and effect on different integrated neural circuits independent of its mechanisms of action (Borsook et al., 2006). We hypothesized that areas associated with the somatosensory cortices and thalamus would be affected in a dose-dependent manner upon GAT107 administration.

## 2. Methods

### 2.1. Animal usage

Male Sprague Dawley rats (*n* = 48) approximately 90 days of age and weighing approximately 400 grams were obtained from Charles River Laboratories (Wilmington, Massachusetts, USA). Rats were maintained on a 12:12 h reverse light–dark cycle with lights off at 07:00 h and allowed access to food and water ad libitum. All rats were acquired and cared for in accordance with the guidelines published in the Guide for the Care and Use of Laboratory Animals (National Institutes of Health Publications No. 85–23, Revised 1985) and adhered to the National Institutes of Health and the American Association for Laboratory Animal Science guidelines. The protocol (#20-0628R) used in this study complied with the regulations of the Institutional Animal Care and Use Committee at Northeastern University and adhered to the ARRIVE guidelines for reporting *in vivo* experiments in animal research (Kilkenny et al., 2010).

### 2.2. Drug preparation and administration

GAT107 was obtained from the Thakur lab at Northeastern University. Solid was dissolved in 1:1:18 ethanol: Emulphor 620: water. To aid in the dissolution process, the solution was heated and sonicated for 20 min, then vortex vigorously for 2–3 cycles or until all solid was dissolved. The solution was stored at 4°C and vortexed vigorously before administration. Rats were randomly assigned to one of four groups corresponding to vehicle (*n* = 6), 1 mg/kg (*n* = 7), 3 mg/kg (*n* = 7), or 10 mg/kg (*n* = 7) GAT107. The dose range was taken from the literature (Bagdas et al., 2016). Drug and vehicle were administered I.P. using a polyethylene tube (PE-50), approximately 30 cm in length, placed in the peritoneal cavity to allow for remote delivery of the drug during imaging.

### 2.3. Awake rat imaging

#### 2.3.1. Imaging system

**FIGURE 1:**
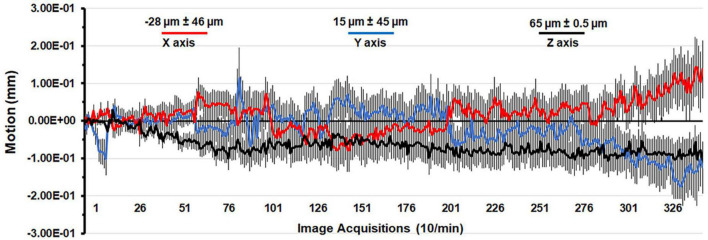
Motion associated with awake imaging. Shown is the degree of motion recorded over the 35 min imaging protocol. The data are reported as the mean and standard error in micrometers for axis X, Y, and Z from all rats from each experimental condition.

A detailed description of the awake rat imaging system is published elsewhere (Stenroos et al., 2018). Notably, we used a quadrature transmit/receive volume coil (ID = 42 mm) that provided both high anatomical resolution and high signal-to-noise ratio for voxel-based BOLD phMRI. The design of the rat holder (Ekam Imaging, Boston, MA) stabilized the head in padded sidebars, minimizing discomfort caused by ear bars and other restraint systems that are commonly used to immobilize the head for awake animal imaging. Shown in Figure 1 is the mean ± SE for the movement (μm) in the x, y, and z axes over the 35 min scanning session for all rats in the study. The average displacement in any orthogonal direction over the entire scanning session did not exceed 65 μm. Rats with repeated motion exceeding 150 μm, i.e., greater than one half the in-plane dimensions of a voxel (ca 300 μm^2^) in any orthogonal direction were excluded from the study. Given the design of the head holder to minimize stress and discomfort with the least mechanical restriction, loss of subjects due to gross motion artifact was unavoidable.

#### 2.3.2. Acclimation

A week prior to the first imaging session, all rats were acclimated to the head restraint and the noise of the scanner. Rats were first secured in the holding system under 1–2% isoflurane anesthesia. The entire procedure usually takes less than 5 min. A video of the rat preparation for imaging is available at www.youtube.com/watch?v=JQX1wgOV3K4. Upon regaining consciousness, rats were placed for up to 45 min in a “mock MRI scanner” environment, consisting of an enclosed black box equipped with an audio recording of MRI pulses. The acclimation protocol was repeated over five consecutive days to reduce autonomic nervous system-induced effects during awake animal imaging (e.g., changes in heart rate, respiration, corticosteroid levels, and motor movements), with the goal of improving contrast-to-noise ratios and image quality (King et al., 2005). There are many published variations on acclimation protocols for preparing rodents for awake imaging (Upadhyay et al., 2011; Russo et al., 2021; Lindhardt et al., 2022).

#### 2.3.3. BOLD phMRI image acquisition and pulse sequence

**FIGURE 2:**
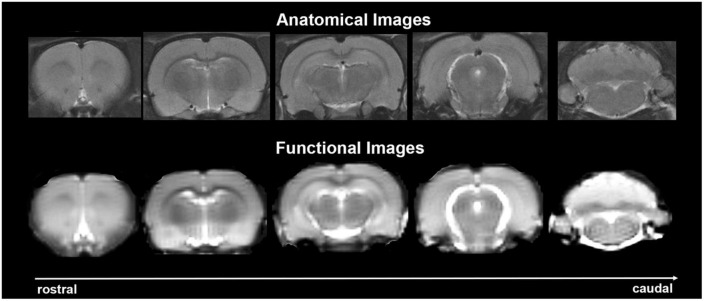
Anatomical fidelity. Shown are representative examples of brain images collected during a single imaging session using a multi-slice spin echo, RARE (rapid acquisition with relaxation enhancement) pulse sequence. The **(top)** shows coronal sections collected during the anatomical scan taken at the beginning of each imaging session. The **(bottom)** row shows the same images but collected for functional analysis using HASTE. These images were acquired using the same field of view and slice anatomy but a larger data matrix of 96 × 96.

Experiments were conducted using a Bruker Biospec 7.0 T/20-cm USR horizontal magnet (Bruker) and a 2 T/m magnetic field gradient insert (ID = 120 mm) capable of a 120-μs rise time. At the beginning of each imaging session, a high-resolution anatomical data set was collected using the rapid acquisition, relaxation enhancement (RARE) pulse sequence (RARE factor 8); (25 slices; 1 mm; field of view (FOV) 3.0 cm^2^; data matrix 256 × 256; repetition time (TR) 3 s; echo time (TE) 12 ms; Effective TE 48 ms; number of excitations (NEX) 3; 4.48 min acquisition time; in-plane resolution 117.2 μm^2^). Functional images were acquired using a multi-slice Half Fourier Acquisition Single Shot Turbo Spin Echo (HASTE) pulse sequence (22 slices; 1.1 mm; FOV 3.0 cm^2^; data matrix 96 × 96; TR 6 s; TE 3.75 ms; Effective TE 22.5 ms; 35 min acquisition time; in-plane resolution 312.5 μm^2^). This spatial resolution is enough to delineate the bilateral habenula (ca 4–5 voxels for each side) but not between lateral and medial habenula. The use of spin echo to acquire the functional images was necessary to achieve the high anatomical fidelity required for data registration to the rat MRI atlas as shown in Figure 2.

Imaging awake rats began ca 15 min after their setup in the restraining system using 1–2% isoflurane. Rats are normally fully ambulatory within 5 min after the cessation of 1–2% isoflurane anesthesia. Each functional imaging session consisted of uninterrupted data acquisitions (whole brain scans) of 350 scan repetitions or acquisitions for a total elapsed time of 35 min. The 5 min baseline included the first 50 image acquisitions, then vehicle or drug administration, followed by another 300 acquisitions over 30 min. The order of drug doses was randomized over the scanning sessions. After the 35 min functional imaging scan, all rats were exposed to 1% isoflurane reducing their rate of respiration to between 50 and 60 breaths/min. This adjustment in respiration took ca 5 min after which functional connectivity images were collected over a 15 min period. Functional connectivity scans were collected using a spin-echo triple-shot EPI sequence (imaging parameters: matrix size = 96 × 96 × 20 (H × W × D), TR/TE = 1,000/15 ms, voxel size = 0.312 × 0.312 × 1.2 mm, slice thickness = 1.2 mm, with 150 repetitions, time of acquisition 15 min.

#### 2.3.4. CO_2_ challenge to assess neurovascular coupling

To test whether the vehicle (1:1:18 ethanol: Emulphor 620: water) had any effect on vascular responsivity that could interfere with the BOLD signal, we challenged male Wistar rats (*n* = 3) with 5% CO2 before and following vehicle administration in the same scanning session. Following a five min baseline (50 images acquisitions) rats were imaged for 5 min (50 image acquisitions) while exposed to 5% CO_2_ through a nasal tube and again for 5 min following cessation of CO_2_. After a delay of 5–10 min, this imaging protocol was repeated but after an IP injection of the vehicle, as described above, at the beginning of the delay period. Imaging lasted for 15 min for each condition and scans were acquired using the HASTE pulse sequence, as described above.

#### 2.3.5. Imaging data analysis

fMRI data analysis was performed in three steps: pre-processing, processing and post-processing. All three steps were accomplished using SPM-12 (https://www.fil.ion.ucl.ac.uk/spm/). Pre-processing involved co-registration, motion correction, smoothing and detrending. Co-registration was done with the following parameters: Quality 0.97, Smoothing 0.6 mm, Separation 0.4 mm. Gaussian smoothing was performed with an FWHM of 0.8 mm. Processing involved registration of the data to rat atlas, followed by segmentation and statistical analysis. For registration and segmentation, all images were first aligned and registered to a 3D Rat Brain Atlas ©with 173 segmented and annotated brain regions with GUI based EVA software (Ekam Solutions, Boston MA). Image registration involved translation, rotation, and scaling, independently and in all three dimensions. All applied spatial transformations were compiled into a matrix [*T*_*j*_] for the *j*th subject. Every transformed anatomical pixel location was tagged with a brain area to generate fully segmented representations of individual subjects within the atlas.

The dose-dependent effect of GAT107 on brain activity was quantified through positive and negative percent changes in BOLD signal relative to baseline. The initial analyses of signal change in individual subjects were done comparing image acquisitions 300–345 to baseline 5–45. The statistical significance of these changes was assessed for each voxel (∼15,000 per subject, in their original reference system) with an independent Student t-tests, with a 1% threshold to account for normal fluctuations of BOLD signal in the awake rodent brain. As a result of the multiple t-tests performed, a false-positive detection controlling mechanism was introduced (Genovese et al., 2002), to ensure that, on average, the false positive detection rate remained below 0.05. The following formula was used:


(1) $$P_{i}\leq \frac{i}{V}\,\frac{q}{c\,\left(V\right)},$$

where *P_i_* is the *p*-value from the t-test at the *i*-th pixel within the region of interest (ROI) containing *V* pixels, each ranked based on its probability value. The false-positive filter value *q* was set to 0.2 for our analysis, and the predetermined constant *c* (*V*) was set to unity (Benjamini and Hochberg, 1995), providing conservative estimates for significance. Pixels that were statistically significant retained their relative percentage change values, while all other pixel values were set to zero. A 95% confidence level, two-tailed distributions, and heteroscedastic variance assumptions were employed for the t-tests.

Composite maps of the percent changes in BOLD signal were built for each experimental group. Each composite pixel location (row, column, and slice) was mapped to a voxel of the *j*th subject by virtue of the inverse transformation matrix [T*j*]^–1^. A trilinear interpolation of subject-specific voxel values determined their contribution to the composite representation. The use of the inverse matrices ensured that the full composite volume was populated with subject inputs. The average of all contributions was assigned as the percent change in BOLD signal at each voxel within the composite representation of the brain for the respective experimental group.

**FIGURE 3:**
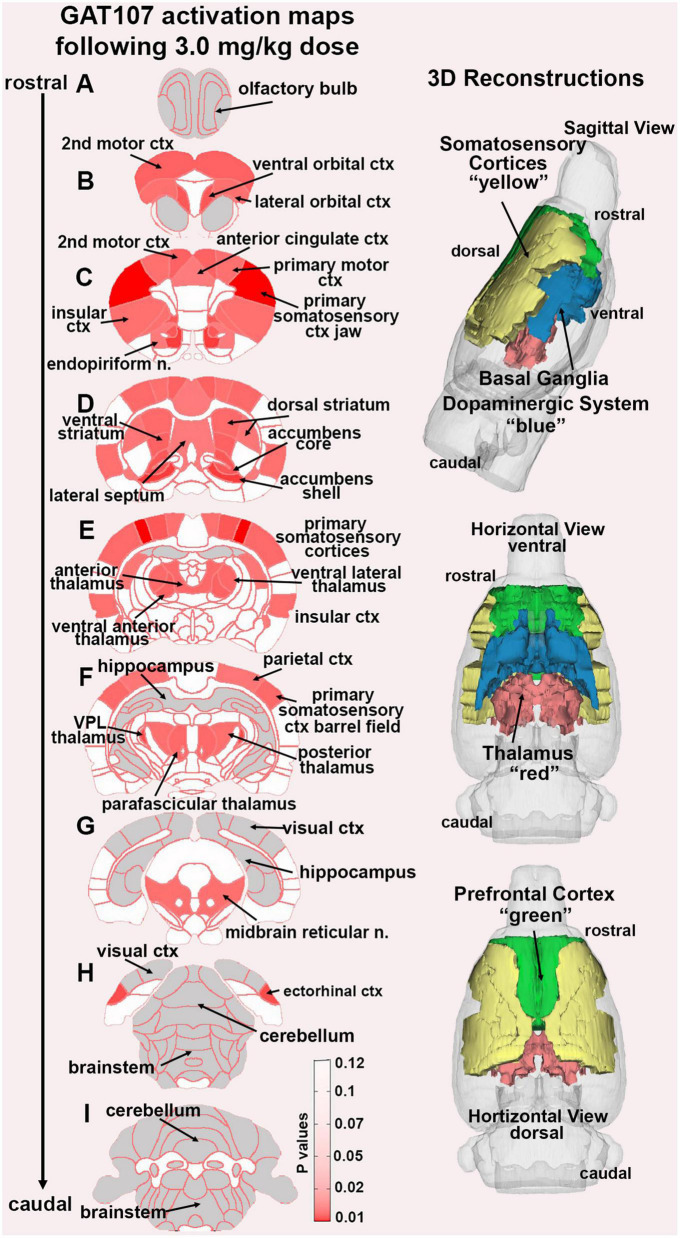
GAT107 statistical heat maps. The 2D coronal sections show the localization of brain areas that were significantly different between vehicle and the 3 mg/kg dose of GAT107. Sections are aligned rostral (top) to caudal (bottom). The color-coded 3D reconstructions to the right are a summary of the major brain regions activated by 3 mg/kg of GAT107.

In the post-processing step, the number of activated voxels in each of the 173 regions was then compared between the control and GAT107 doses using a Kruskal–Wallis test statistic. The data were ranked in order of significance as shown in Table 1. Probability heat maps as shown in Figure 3, were generated showing brain areas of significant differences when compared between two or more groups. The saturation of pink color indicates lower *p*-value (higher confidence) for the brain area.

**TABLE 1:** Dose response.

	Veh	1.0 mg	3.0 mg	10.0 mg	
**Brain area**	**Med**	**Med**	**Med**	**Med**	***P* val**
**Positive BOLD volume of activation**					
Primary somatosensory ctx trunk	0	0	17	0	0.008
Precuniform nucleus	0	0	1	0	0.013
1st cerebellar lobule	0	5	0	8	0.014
Motor trigeminal nucleus	2	0	4	2	0.016
Primary somatosensory ctx jaw	22	33	160	2	0.017
Supraoptic nucleus	1	0	0	1	0.019
Ventral posteriolateral thalamus	3	0	27	5	0.021
Primary motor ctx	34	32	275	15	0.022
Raphe linear	0	0	5	0	0.026
Primary somatosensory ctx shoulder	0	3	14	0	0.026
Root of trigeminal nerve	33	13	35	57	0.027
Premammillary nucleus	0	0	4	3	0.028
Median raphe nucleus	0	0	11	0	0.030
Infralimbic ctx	17	6	34	24	0.030
Endopiriform nucleus	2	3	26	0	0.031
Accumbens core	1	0	25	0	0.032
Paraflocculus cerebellum	84	28	54	121	0.033
Pineal gland	0	3	0	0	0.035
White matter	110	98	261	163	0.035
Insular ctx	42	42	203	26	0.035
Ventrolateral thalamus	0	1	15	0	0.037
Parvicellular reticular nucleus	36	17	29	68	0.039
Primary somatosensory ctx hindlimb	1	8	67	0	0.040
Claustrum	0	0	7	0	0.043
Parafascicular thalamus	4	0	42	5	0.045
Secondary motor ctx	15	27	118	10	0.047
Anterior thalamus	3	0	36	3	0.047
Reticular nucleus midbrain	28	30	82	40	0.051
Pontine reticular nucleus oral	3	1	44	4	0.052
CA3 dorsal	13	16	58	31	0.052
Pontine reticular nucleus caudal	7	1	20	3	0.054
Parietal ctx	3	0	33	3	0.057
Prelimbic ctx	14	2	59	11	0.058
Ventral anterior thalamic nucleus	0	0	10	0	0.059

Post-hoc analyses were performed with a Wilcoxon rank-sum test. Brain regions were considered statistically different between experimental groups when *p* ≤ 0.05. The data from the combined somatosensory cortices was also analyzed as a percent change in BOLD signal over time following the injection of the 3 mg/kg dose GAT107 or vehicle. The signal at each time point (350 image acquisition) from all seven rats at this dose or vehicle (*n* = 6) was averaged and plotted as a time course and analyzed using a two-way repeated measures ANOVA using GraphPad Prism version 9.0.0 (86) for macOS, GraphPad Software, San Diego, California USA, www.graphpad.com.

### 2.4. Resting state functional connectivity

#### 2.4.1. Image acquisition

Scans were collected using a spin-echo triple-shot EPI sequence (imaging parameters: matrix size = 96 × 96 × 20 (H × W × D), TR/TE = 1,000/15 ms, voxel size = 0.312 × 0.312 × 1.2 mm, slice thickness = 1.2 mm, with 200 repetitions, time of acquisition 15 min. Preprocessing was accomplished by combining Analysis of Functional NeuroImages (AFNI_17.1.12, http://afni.nimh.nih.gov/afni/), FMRIB Software library (FSL, v5.0.9, http://fsl.fmrib.ox.ac.uk/fsl/), Deformable Registration via Attribute Matching and Mutual-Saliency Weighting (DRAMMS 1.4.1, https://www.cbica.upenn.edu/sbia/software/dramms/index.html) and MATLAB (Mathworks, Natick, MA). Brain tissue masks for resting-state functional images were manually drawn using 3DSlicer (https://www.slicer.org/) and applied for skull-stripping. Motion outliers (i.e., data corrupted by extensive motion) were detected in the dataset and the corresponding time points were recorded so that they could be regressed out in a later step. Functional data were assessed for the presence of motion spikes. Any large motion spikes were identified and removed from the time-course signals. This filtering step was followed by slice timing correction from interleaved slice acquisition order. Head motion correction (six motion parameters) was carried out using the first volume as a reference image. Normalization was completed by registering functional data to the 3D MRI Rat Brain Atlas ©using affine registration through DRAMMS. The MRI rat atlas containing 173 annotated brain regions was used for segmentation. After quality assurance, band-pass filtering (0.01 Hz∼0.1 Hz) was preformed to reduce low-frequency drift effects and high-frequency physiological noise for each subject. The resulting images were further detrended and spatially smoothed (full width at half maximum = 0.8 mm). Finally, regressors comprised of motion outliers, the six motion parameters, the mean white matter, and cerebrospinal fluid time series were fed into general linear models for nuisance regression to remove unwanted effects.

The region-to-region functional connectivity method was performed to measure the correlations in spontaneous BOLD fluctuations. A network is comprised of nodes and edges; nodes being the brain region of interest (ROI) and edges being the connections between regions. Voxel time series data were averaged in each node based on the residual images using the nuisance regression procedure. Pearson’s correlation coefficients across all pairs of nodes (14535 pairs) were computed for each subject among all three groups to assess the interregional temporal correlations. The r-values (ranging from –1 to 1) were z-transformed using the Fisher’s Z transform to improve normality. 166 × 166 symmetric connectivity matrices were constructed with each entry representing the strength of edge. Group-level analysis was performed to look at the functional connectivity in the experimental groups. The resulting Z-score matrices from one-group t-tests were clustered using the K-nearest neighbors clustering method to identify how nodes cluster together and form resting state networks. A Z-score threshold of | Z| = 2.3 was applied to remove spurious or weak node connections for visualization purposes.

#### 2.4.2. Functional connectivity analysis

##### 2.4.2.1. Degree centrality

All network analysis was computed with Gephi, an open-source network analysis and visualization software (Bastian et al., 2009). Absolute values of the GAT107 and vehicle symmetric connectivity matrices were imported, and edges were loaded as undirected networks. Degree centrality analysis quantifies the number of connections a specific node has to the overall network. Degree centrality is defined as:


$${\text{C}}_{\text{D}}\,\left(j\right)=\sum_{\text{j}=1}^{n}{\text{A}}_{\text{ij}}$$

where *n* is the number of rows in the matrix in the adjacency matrix A and the elements of the matrix are given by A_ij_, the number of edges between nodes i and j.

##### 2.4.2.2. Statistics

All statistical analysis for the graph theory analysis was performed using GraphPad Prism. Normality tests between group subregions were performed to determine if parametric or non-parametric assumptions were needed. Shapiro-Wilk’s tests were performed to analyze normality assumption. Subregion degree centrality *p*-values that were greater than 0.05 were assumed to be normal. After assumptions of normality were validated, paired t-tests were used to compare degree centrality of the GAT107 and vehicle groups in various subregions. When necessary, a nonparametric Wilcoxon signed rank (WSR) test is performed if there is evidence against the normality assumption.

## 3. Results

### 3.1. Positive BOLD volume of activation

Shown in Table 1 is a truncated list of brain areas showing the median (Med) number of voxels activated for vehicle (Veh) and each dose of GAT107 together with their *p* value. With only a few exceptions, the 3 mg/kg dose of GAT107 (highlighted in gray) was higher than vehicle, 1 and 10 mg/kg doses of GAT107 resulting in an inverted U-shape dose-response. This pattern of activity is present in a majority of the 173 brain areas comprising the rat MRI atlas as shown in Supplementary Table 1. A post hoc Wilcoxon rank-sum test identified 38/173 brain areas with a significant increase in the positive volume of activation i.e., voxel number, in response to the 3 mg/kg dose of GAT107. These brain areas are listed in Table 2 and ranked in order of their significance using a critical value of *p* < 0.05 but taking into consideration a false discovery rate of *p* = 0.034. Note the representation of the primary somatosensory cortices (e.g., shoulder, trunk, jaw, hindlimb, barrel field and forelimb). A complete table of all brain areas comparing vehicle to the 3 mg/kg dose is provided in Supplementary Table 2.

**TABLE 2:** Vehicle vs 3.0 mg/kg GAT107.

	Veh		3 mg/kg			
**Brain area**	**Med**		**Med**	***P*-val**	**Ω Sq**	
**Positive BOLD volume of activation**						
Primary somatosensory ctx shoulder	0	<	14	0.013	0.495	
Primary somatosensory ctx trunk	0	<	17	0.013	0.493	
Primary somatosensory ctx jaw	22	<	160	0.016	0.463	
Accumbens shell	15	<	33	0.020	0.420	
Precuniform nucleus	0	<	1	0.021	0.412	
Median raphe nucleus	0	<	11	0.021	0.411	
Ectorhinal ctx	7	>	0	0.022	0.402	
Ventral posteriolateral thalamic nucleus	3	<	27	0.024	0.389	
Ventral orbital ctx	2	<	36	0.027	0.366	
Accumbens core	1	<	25	0.028	0.032	
Primary somatosensory ctx hindlimb	1	<	67	0.028	0.364	
Ventral anterior thalamic nucleus	0	<	10	0.030	0.353	
Secondary motor ctx	15	<	118	0.030	0.049	
Ventral medial striatum	0	<	56	0.031	0.346	
Premammillary nucleus	0	<	4	0.032	0.021	
Ventrolateral thalamic nucleus	0	<	15	0.033	0.337	
Anterior thalamic nuclei	3	<	36	0.036	0.320	
Dorsal medial striatum	4	<	123	0.036	0.320	
Parafascicular thalamic nucleus	4	<	42	0.036	0.320	
Primary somatosensory ctx barrel field	27	<	156	0.036	0.320	
Primary somatosensory ctx forelimb	7	<	123	0.036	0.320	
Lateral orbital ctx	11	<	59	0.037	0.316	
Reticular nucleus midbrain	28	<	82	0.037	0.022	
Insular ctx	42	<	203	0.037	0.314	
Primary motor ctx	34	<	275	0.037	0.314	
Posterior thalamic nucleus	8	<	46	0.037	0.314	
White matter	110	<	261	0.037	0.037	
Dorsal lateral striatum	5	<	124	0.042	0.297	FDR
Pontine reticular nucleus oral	3	<	44	0.043	0.003	*p* = 0.039
Parietal ctx	3	<	33	0.043	0.039	
Endopiriform nucleus	2	<	26	0.045	0.285	
Anterior cingulate ctx	19	<	116	0.045	0.059	
Lateral septal nucleus	22	<	89	0.045	0.284	

Figure 3 shows the anatomical localization of the brain areas listed in Table 1 presented as statistical heat maps. The coronal sections are labeled A-I and arranged from rostral (top) to caudal (bottom). Brain section (A), representing the olfactory bulbs highlighted in gray is devoid of activity as are the most caudal sections (H) and (I) representing the brainstem and cerebellum with the exception of the ectorhinal ctx. Note the many brain areas comprising the prefrontal cortex in sections (B) and (C) (e.g., 2nd motor ctx, ventral and lateral orbital cortices, and anterior cingulate ctx). The efferent connections of the midbrain dopaminergic system are displayed in section (D) (e.g., accumbens shell and core, lateral septum, dorsal, and ventral striatum). The thalamus is represented in sections (E) and (F) together with the many areas of the primary somatosensory cortices as noted in Table 1. It should be noted that the hippocampal complex (sections E-G) and visual cortex (sections G-H) highlighted in gray were also devoid of any activity. The 3D color coded reconstructions to the right summarize the major brain areas activated by the 3 mg/kg dose of GAT107.

### 3.2. Percent change in BOLD signal over time in the somatosensory cortex

**FIGURE 4:**
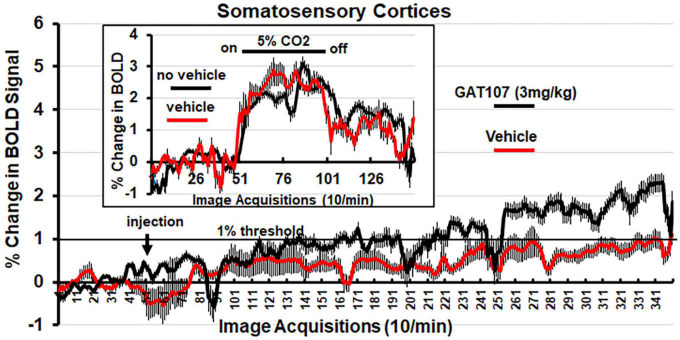
Change in BOLD signal over time. Percent positive BOLD change over time at the most efficacious dose, 3 mg/kg GAT107, in the somatosensory cortices. The insert shows the percent positive BOLD change over time in the somatosensory cortices when challenged with 5% CO_2_ with and without vehicle to assess neurovascular coupling.

Figure 4 shows the percent change in BOLD signal over time following injection (arrow) of vehicle or 3 mg/kg dose of GAT107. The mean ± SE for each of the 350-image acquisition is the composite of all primary somatosensory cortical areas listed in Table 1. The 1% threshold represents the level of background noise typical in awake animal imaging. A two-way, repeated measures ANOVA found a significant (*p* < 0.0001) interaction between drug X time. A Sidak’s multiple comparison test found a majority of the time points at acquisitions 286–340 were significantly different. Although GAT107 significantly elevated BOLD signal, we were surprised that the change was only two percent and not the typical 4–6% reported in other studies (Ferris et al., 2015). To test whether our vehicle (comprised of 5% ethanol and an uncommon emulsifier, Emulphor620) affected vascular responsivity, we performed a 5% CO2 challenge with and without vehicle on the same rat (*n* = 3) during their scanning session. The results are shown in the insert of Figure 4 and again are a composite of all primary somatosensory cortical areas. There was no significant difference between the change in BOLD signal with and without vehicle. Both conditions showed a modest change in BOLD signal between 2 and 3% suggesting this is true for the primary somatosensory cortices under these experimental conditions (Ferris et al., 2014). When the primary olfactory system was analyzed for its percent change in BOLD signal to 5% CO2 challenge the changes were between 4 and 6% (see Supplementary Figure 1).

### 3.3. Resting state functional connectivity

**FIGURE 5:**
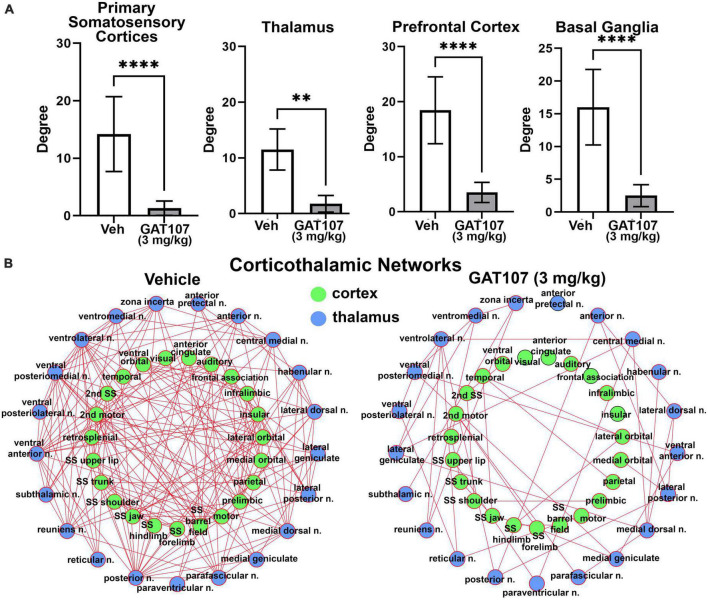
Functional connectivity. **(A)** The number of degrees for the four main brain regions: primary somatosensory cortices, thalamus, prefrontal cortex, and basal ganglia. **(B)** Corticothalamic neural network. **p < 0.01; ****p < 0.0001.

The bar graphs in Figure 5A report the mean ± SD for the number of degrees (hubs or brain areas) for the four main brain regions highlighted in the 3D reconstructions in Figure 3, i.e., primary sensory motor cortices, thalamus, prefrontal cortex and basal ganglia. In all cases the vehicle treatment is significantly greater than 3 mg/kg GAT107. The sensorimotor cortices and prefrontal cortex and their connectivity with the thalamus is shown in Figure 5B below as a corticothalamic neural network. The hypoconnectivity caused by GAT107 treatment is remarkable when compared to the many edges (connections) shown as red lines in the vehicle neural network.

## 4. Discussion

GAT107 is a novel ago-PAM designed to modulate the α7 nAChR function. Previously reported studies on GAT107 have focused on ion channel conductance in oocytes and behavioral models of inflammation and pain (Bagdas et al., 2016; Gauthier et al., 2021; Mizrachi et al., 2021). The present study used phMRI to evaluate the dose-dependent effect of GAT107 on brain activity in awake male rats. phMRI is an imaging modality used to identify a drug’s effect on global brain activity and connectivity independent of its mechanism of action (Borsook et al., 2006). In this case, GAT107 presented with an inverted-U dose response with selective activity in the forebrain, sensory motor cortex and thalamus that together showed a significant decrease in neural network connectivity.

Electrophysiology studies have shown that GAT107 acts as a PAM in the presence of acetylcholine and an agonist in its absence at the α7nAChR (Papke et al., 2014; Quadri et al., 2019). Molecular modeling and mutagenesis studies (Papke et al., 2014; Horenstein et al., 2016) have also shown that GAT107 binds to a site distinct from the orthosteric site along a region of aromatic amino acids inside the receptor pore. Additionally, electrophysiology subtype selectivity studies at a range of human recombinant nAChRs have shown that 4BP-TQS, the unresolved racemate of GAT107, has selectivity for the α7 nAChRs (Gill et al., 2013). However, even though 4BP-TQS does not act as an agonist or PAM at any other nAChRs, GAT107 has not undergone any additional off-target studies.

α7 nAChRs receptors are highly expressed in the hippocampus, thalamus, somatosensory cortices, prefrontal cortex, and reticular formation (Alkondon et al., 2000; Lambe et al., 2003; Martins and Tavares, 2017) predominantly on presynaptic GABAergic interneurons and glutamatergic neurons (Alkondon et al., 2000; Buhler and Dunwiddie, 2002). The pattern of positive BOLD activation mirrored many of these areas with a high density of α7 nAChRs. However, GAT107 had no significant effect on BOLD signal in the hippocampus despite a high receptor concentration in this region. α7 nAChRs are expressed on GABAergic interneurons in the hippocampus and may function to reduce hippocampal activity which can dampen changes in BOLD signal. However, it’s important to note that the α7 nAChRs are expressed on both GABAergic interneurons and glutamatergic neurons and regulate neurotransmission in the hippocampus (Alkondon et al., 1997; Buhler and Dunwiddie, 2002), making it difficult to explain the absence of BOLD signal in this area of the brain.

One theory of schizophrenia suggests the thalamus is the key area of dysfunction (Jiang et al., 2021). Disruption in thalamic connections to the somatosensory cortex are responsible for many of the symptoms of this mental illness (Woodward et al., 2012) and patients with chronic schizophrenia present with an increase in functional connectivity to the somatomotor and somatosensory cortices (Giraldo-Chica and Woodward, 2017). The positive symptoms, e.g., delusions and hallucinations, can be controlled in a majority of patients by typical and atypical antipsychotic affecting dopaminergic and serotoninergic neurotransmission. However, there are fewer options for treating the negative symptoms that are characterized by a decrease in affect, motivation, and cognition. α7 nAChR agonists have been reported to restore the cognitive performance disrupted in preclinical models of schizophrenia (Wallace et al., 2011; Brooks et al., 2012; Jones et al., 2014). Nikiforuk et al., reported an α7 PAM, but not an α7 ago-PAM like GAT107, could reverse the cognitive dysfunction and asocial behavior produced in a ketamine model of schizophrenia in rats (Nikiforuk et al., 2016). However, clinical studies with α7 nAChR agonists have fallen short of expectations; having little effect on the cognitive problems faced by patients during the prodromal or negative symptom phase of schizophrenia (Recio-Barbero et al., 2021). In our study using healthy “normal” male rats GAT107 had a dramatic effect on functional connectivity as compared to vehicle treatment. The corticothalamic neural network showed a significant decrease in connectivity following GAT107 treatment. Would change in functional connectivity be accompanied by a positive therapeutic outcome in a rat ketamine model of schizophrenia?

In a previous publication, we reported GAT107 was antinociceptive in a complete Freund’s adjuvant model of neuropathic pain (Bagdas et al., 2016). This was not surprising as nAChR agonists, such as nicotine, have been used for pain relief since the 1,500s (Umana et al., 2013). There is a high concentration of α7 nAChRs in both the spinothalamic tract, responsible for pain modulation (Al-Chalabi et al., 2022), and in the cortico-striatal-thalamo-cortical (CSTC) pathway, responsible for movement control, reinforcement, and sensory perception (Rădulescu et al., 2017). There is clear anatomical and electrophysiological evidence showing nociceptive fibers emanating from lamina1 of the dorsal horn of the spinal cord and their multisynaptic connections to the somatosensory cortex and forebrain (Cechetto et al., 1985; Bernard et al., 1994; Bourgeais et al., 2001). Functional imaging in awake rodents have identified many of these same areas following capsaicin injection into the hind paw during the scanning session (Yee et al., 2015). GAT107 treatment shows activation of the neural pain circuitry, including the somatosensory cortex, ventral posterior lateral thalamus, raphe, insular, and anterior cingulate cortices. However, brainstem and pontine areas associated with the ascending reticular activating system and pain such as the gigantocellularis nucleus, parabrachial nucleus and the periaqueductal gray were not affected by GAT107.

It should also be noted that previous studies suggest that nicotine increases dopamine release in the nucleus accumbens by enhancing excitatory glutamatergic input to the ventral tegmentum area and striatum via presynaptic α7 nAChRs (Kaiser and Wonnacott, 2000; Barik and Wonnacott, 2006). Accordingly, GAT107 activates both the accumbens core and shell (Table 1) as well as the ventral and dorsal medial striatum. However, no significant BOLD activation was seen in the ventral tegmental area, which suggest GAT107’s effect on the dopaminergic pathway is only in projected regions.

### 4.1. Data interpretation and limitations

Any new drug in early discovery can be screened using phMRI in awake animals as a means of assessing dose-dependent changes in brain activity, putative target engagement and global connectivity. The study should be agnostic, without any preconceived notion of what the drug should do to the brain and how it does it. Let the data tell the story. In this case we felt obligated to discuss GAT107 in the context of schizophrenia given the large amount of literature on the development and testing of α7 nAChRs agonists as neuroleptics. This study would have benefited if GAT107 had been tested in behavioral models of psychosis, cognitive and emotional dysfunction.

The global hypoconnectivity was unexpected and inexplicable. Unfortunately, we only ran connectivity analysis on the 3 mg/kg dose of GAT107 for what would seem to be an obvious reason but a positive control like a common neuroleptic or negative data from either the low or high dose of GAT107 would have helped to understand the hypoconnectivity. A thorough review of the animal literature shows there are only two studies testing antipsychotics, e.g., haloperidol, risperidone and olanzapine for their effect on functional connectivity (Gass et al., 2013; Tollens et al., 2018). The studies were done in “healthy “rats under medetomidine anesthesia and were focused on the forebrain. Haloperidol decreased connectivity in all areas studied as compared to vehicle while the atypical antipsychotics with anti- serotonergic activity increased connectivity. These cases do not offer corroborating evidence in support of GAT107 and global hypoconnectivity but do serve to highlight the need for more research in this area.

Another major limitations of our study is the use of only male rats. Indeed, most of the studies on GAT107 were conducted in male mice. There is a sex difference in the CNS nAChR, specifically in the reward system (Giraldo-Chica and Woodward, 2017; Jiang et al., 2021), which could have influenced the pattern of activation and magnitude of BOLD caused by GAT107. Another limitation was the lack of pharmacokinetic data to determine the proper duration of imaging, as it was only at the very end of the 30 min imaging session that we recorded a significant change in BOLD signal. A longer scanning time may have presented a greater change in BOLD signal. Further studies on the pharmacokinetic properties of GAT107 would be useful to optimize experimental parameters. To this point, the dose range (1.0, 3.0, and 10 mg/kg) was taken from the literature and may not encompass the full neurobiological effects of GAT107. The inverted U-shape presents a narrow range of activity and as such, may have limited use in the clinic. Still another limitation was the collection of functional connectivity data under light isoflurane anesthesia to minimize motion and physiological stress (Gorges et al., 2017). What constitutes a “resting state” condition in a head restrained, awake rodent could be debated. Anesthesia may reduce the magnitude of the BOLD signal but does not disrupt the connectivity as demonstrated across species and under different physiological conditions (Vincent et al., 2007; Liang et al., 2012; Guilfoyle et al., 2013; Jonckers et al., 2014; Gozzi and Schwarz, 2016). As a point of caution, GAT107 activates accumbens and ventral striatum, brain areas associated with reward seeking behavior. Future studies should test the possibility that GAT107 has abuse liability.

### 4.2. Summary

This study was performed to examine the global effects of GAT107 on brain areas associated with schizophrenia and pain. The rapid and widespread effects of GAT107 were assessed using phMRI in healthy, awake male rats. There was an inverted U dose-response, with the 3 mg/kg dose being the most efficacious. GAT107 activates specific brain regions involved in cognitive control, motivation, and sensory perception. GAT107 causes widespread hypoconnectivity.

## Data availability statement

The original contributions presented in this study are included in the article/Supplementary material, further inquiries can be directed to the corresponding author.

## Ethics statement

The protocol (#20-0628R) used in this study complied with the regulations of the Institutional Animal Care and Use Committee at Northeastern University.

## Author contributions

BB, ES, JC, PK, and CF: experimental design and manuscript preparation. BB, ES, JC, JZ, and PK: data generation and analysis. AC and RO: functional connectivity analysis. LC and GT: compound synthesis and provided the compound. All authors contributed to the article and approved the submitted version.
