# Supplementary figures and images for: Dose-dependent effects of GAT107, a novel allosteric agonist-positive allosteric modulator (ago-PAM) for the α7 nicotinic cholinergic receptor: a BOLD phMRI and connectivity study on awake rats
Source: Front Neurosci. 2023 Jun 23;17:1196786. doi: 10.3389/fnins.2023.1196786 (PMC10326388; doi:10.3389/fnins.2023.1196786)

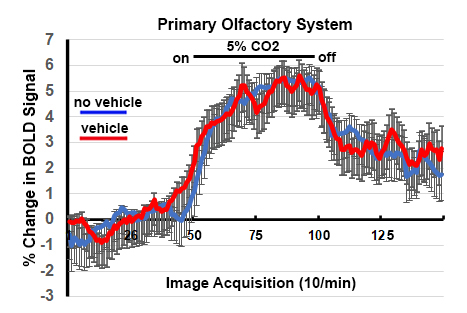

Supplement: Supplementary file 3 [file Image_1.JPEG]
